# Centre of Rotation of the Human Subtalar Joint Using Weight-Bearing Clinical Computed Tomography

**DOI:** 10.1038/s41598-020-57912-z

**Published:** 2020-01-23

**Authors:** Marta Peña Fernández, Dorela Hoxha, Oliver Chan, Simon Mordecai, Gordon W. Blunn, Gianluca Tozzi, Andy Goldberg

**Affiliations:** 1 Zeiss Global Centre, School of Mechanical and Design Engineering, University of Portsmouth, Portsmouth, PO1 3DJ UK; 2 UCL Institute of Orthopaedics & Musculoskeletal Science, Division of Surgery & Interventional Science, Royal National Orthopaedic Hospital, Stanmore, HA7 4LP UK; 3 School of Pharmacy and Biomedical Sciences, University of Portsmouth, Portsmouth, PO1 2DT UK; 4 MSK Lab, Faculty of Medicine, Department of Surgery & Cancer, Imperial College London, Level 2, Faculty Building, South Kensington Campus, London, SW7 2AZ UK; 5 The London Ankle & Arthritis Centre, The Wellington Hospital, Wellington Place, London, NW8 9LE UK

**Keywords:** Bone, Biomedical engineering

## Abstract

Accurate *in vivo* quantification of subtalar joint kinematics can provide important information for the clinical evaluation of subtalar joint function; the analysis of outcome of surgical procedures of the hindfoot; and the design of a replacement subtalar joint prosthesis. The objective of the current study was to explore the potential of full weight-bearing clinical computed tomography (CT) to evaluate the helical axis and centre of rotation of the subtalar joint during inversion and eversion motion. A subject specific methodology was proposed for the definition of the subtalar joint motion combining three-dimensional (3D) weight-bearing imaging at different joint positions with digital volume correlation (DVC). The computed subtalar joint helical axis parameters showed consistency across all healthy subjects and in line with previous data under simulated loads. A sphere fitting approach was introduced for the computation of subtalar joint centre of rotation, which allows to demonstrate that this centre of rotation is located in the middle facet of the subtalar joint. Some translation along the helical axis was also observed, reflecting the elasticity of the soft-tissue restraints. This study showed a novel technique for non-invasive quantitative analysis of bone-to-bone motion under full weight-bearing of the hindfoot. Identifying different joint kinematics in patients with ligamentous laxity and instability, or in the presence of stiffness and arthritis, could help clinicians to define optimal patient-specific treatments.

## Introduction

**Figure 1:**
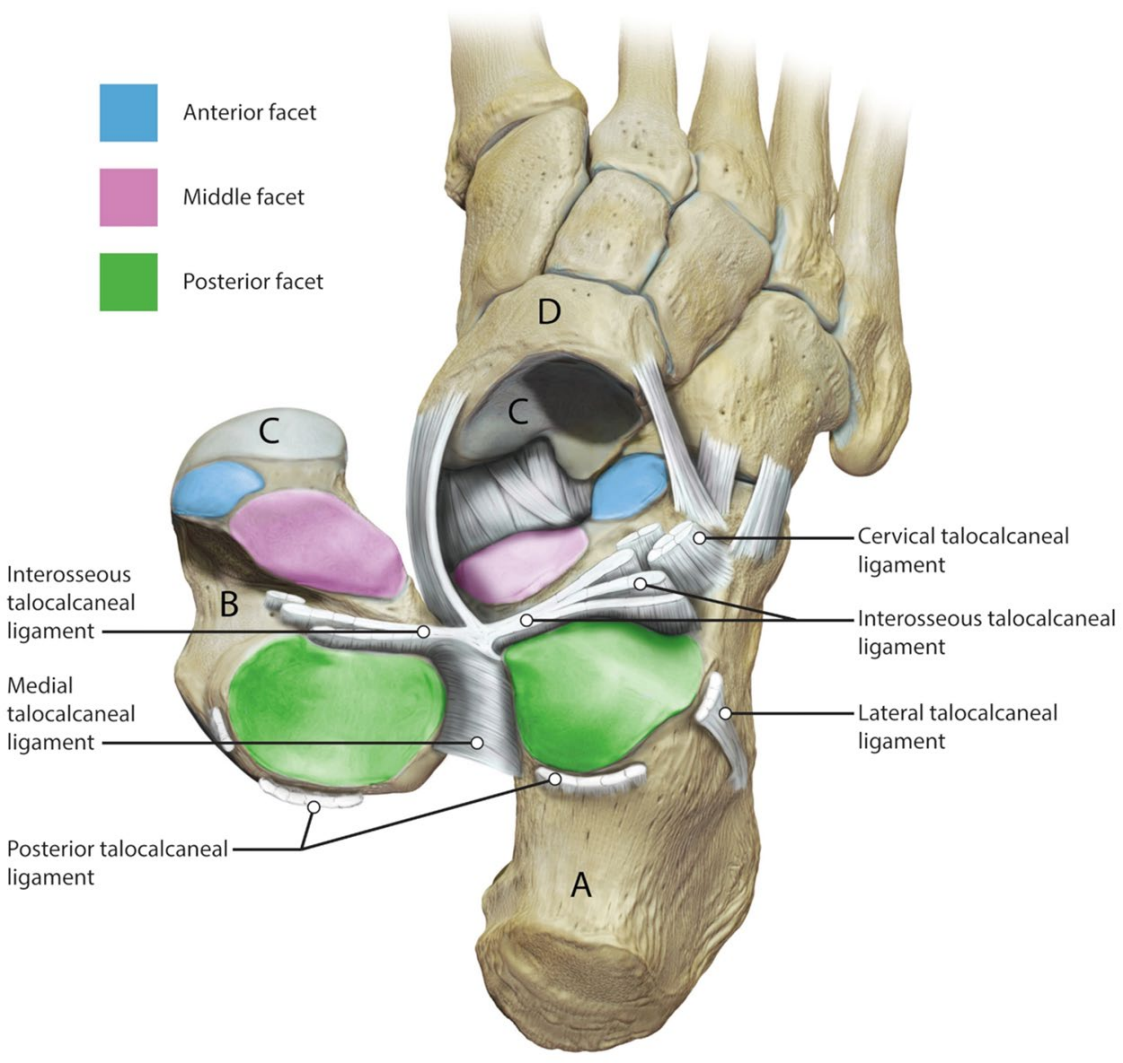
An illustration of the subtalar joint. The ligaments on the outside of the joint have been divided and the talus (**B**) has been reflected. The calcaneus (**A**) is visible from above. The three articular facets of the subtalar joint are illustrated, the posterior facet (green); the middle facet (pink) and the anterior facet (blue). The head of the talus articulates with the navicular bone (**D**) anteriorly at the talonavicular joint (**C**). The soft tissue ligamentous restraints are labelled. Image by Catherine Sulzmann, Medical Artist.

The subtalar joint describes an articulation between talus and calcaneus, forming one of two joints of the hindfoot with the tibiotalar or ankle joint above the talus and the subtalar joint below. The talus comprises of three facets (anterior, middle and posterior) that articulate with the mating facets on the calcaneus at the subtalar joint. The bones are connected by a complex of ligamentous structures that connect the talus to the calcaneus and both structures to the adjacent navicular bone, which is intricately involved in hindfoot and midfoot motion (Fig. 1). The subtalar joint is the primary joint involved in motion and posture of the hindfoot in the frontal plane. Motion is complex and it combines dorsiflexion, abduction and eversion in one direction and plantarflexion, abduction and inversion in the other^1^.

Problems associated to the subtalar joint can have a significant impact on function^2^, preventing participation in sports and normal daily activities. Common pathologies affecting the joint include instability following ligamentous injury^3^, and painful flat feet in children and adults. In addition, end stage ankle and hindfoot arthritis is a major problem that has been shown to affect quality of life as much as end stage heart disease^4,5^. Although the incidence of subtalar joint arthritis is unknown, approximately 3.4% of the population aged over 50 has radiographic hindfoot arthritis, which is more than 300,000 people in the UK^6^. The most common cause of subtalar joint arthritis is posttraumatic, occurring in almost all patients following a talar body or calcaneal fracture^7,8^. Other non-traumatic causes include longstanding flat feet, tendon dysfunction and inflammatory conditions such as rheumatoid arthritis^9^. Following failure of non-operative management which includes the use of painkillers, ankle supports, and activity modification, the gold standard treatment for end stage subtalar joint arthritis at present consists in the removal of the subtalar joint and fusion of the talus to the calcaneus (subtalar joint fusion) using metalwork. In this situation the talocalcaneal and or talonavicular motion is obliterated and the hindfoot moves as one structure. Fusion causes transmission of abnormal stresses to adjacent joints^10–13^, leading to their increased risk of wear and tear. Although subtalar joint replacement has been attempted, early failure rates greater than 50% within the first year^14^ led to the abandonment of the procedure. The creation of a subtalar joint replacement, that is as functionally effective and as reliable as other joint (i.e. hip and knee) replacements, is inhibited by the higher complexity of the subtalar joint as well as due to lack of understanding of the mechanics of the joint.

Motion of the subtalar joint is complex. Due to the convex posterior facet of the calcaneus and corresponding concave facet of the talus, subtalar joint movement can be described as rotation, translation or a combination of both^1,15^. In general, subtalar joint motion has been described using a finite helical axis. At each moment in time, motion of the subtalar joint can be broken down into a rotation about, and a translation along the helical axis, although it has been shown that the physiological axis cannot be described that simplistically^16–18^. Subtalar joint helical axis is directed obliquely from posterior-lateral-plantar to anterior-medial-dorsal, piercing the talus anteriorly at the superior aspect of the talar neck^19,20^. During weight-bearing motion, the foot has been proposed to behave as a rigid unit with all the bones of the foot rotating around the central pivot of the talus at the subtalar joint axis^19^. The ability to accurately determine the centre of rotation (i.e. central pivot) of human joints is particularly important in the field of orthopaedics, where treatments involve the replacement of joints that need to replicate the native kinematics. Whilst considerable attention has been given to the determination of the centre of rotation of the hip joint^21,22^, knee joint^23,24^ and ankle joint^25,26^, the location of the centre of rotation of the subtalar joint has not been previously identified.

Tracking subtalar joint motion is technically challenging, thus several methodologies have been proposed to study subtalar joint kinematics *in vivo*. External surface markers^17,18,27,28^, dual fluoroscopic imaging^29–31^ and three-dimensional (3D) imaging methods (i.e. computed tomography (CT) and magnetic resonance imaging (MRI))^16,32–35^ have been proposed to address subject-specific subtalar joint biomechanics. Although camera registration techniques provide subtalar joint kinematics during stance and walking, the lack of external landmarks of the talus hinder rotation and translation measurements. Conversely, imaging methods directly measure helical axis of rotation, providing more accurate measurements *in vivo*^16,34,35^. Fluoroscopic imaging overcomes some of these challenges in using external marker-based models, and when coupled with a CT or MRI it can provide 3D information of the joint motion. However, its application for the evaluation of subtalar joint helical axis has not been reported. Recent studies have shown the potential of four-dimensional (4D) CT to evaluate subtalar joint kinematics and detect changes between healthy and pathological subjects both *ex vivo*^36^ and *in vivo*^37^. However, all previous literature reports images acquired with subjects placed in the CT scanner table in a supine position and, although custom-built ankle loading devices are commonly used, images acquired are not in a weight-bearing configuration.

During weight-bearing, the combination of tensile loading of the ligaments and compression loading of all the joints of the foot creates a more tightly joined and stable architecture when compared to a non-weight-bearing situation^19^. Consequently, motion and bone position of the subtalar joint may not be accurately defined with traditional non-weight-bearing imaging techniques. Recently, full weight-bearing standing CT devices such as PedCAT (Curvebeam, Warrington, USA) have been developed, allowing more functional diagnosis of foot problems^38,39^ as well as more accurate measurements of bone alignment^40–43^. PedCAT dual scanning modality (3D functional imaging in full weight-bearing) in combination with the advancements of image-based analysis such as digital volume correlation (DVC)^44^ can be exploited to analyse subtalar joint movement *in vivo*. DVC is a 3D image registration technique able to provide full-field displacement throughout the interior of materials subjected to incremental loading whilst simultaneously imaged (i.e. via CT) and it has been widely used in bone biomechanics^45–47^. The fundamentals of DVC method rely on the measurement of a discrete displacement vector field by correlation of 3D images in different deformation states, based on the natural variations in image grey-level intensity, which are given by the internal material texture of the sample. Thus, a 3D image-based patient specific procedure can be defined to describe subtalar joint motion under full weight-bearing by computing 3D full-field displacement of the subtalar joint from inversion to eversion position.

Therefore, the aim of this study is two-fold. Firstly, a novel method enabling the quantification of inversion-eversion at the subtalar joint based on 3D imaging with full weight-bearing in combination with 3D full-field DVC-computed displacement is introduced. Under the hypothesis that there is a single axis of rotation of the posterior facet of the subtalar joint in normal healthy individuals the second aim of this study is to establish whether there is a centre of rotation of the subtalar joint, effectively a central pivot of the talus along the helical axis.

## Results

### 3D subtalar joint motion

**Figure 2:**
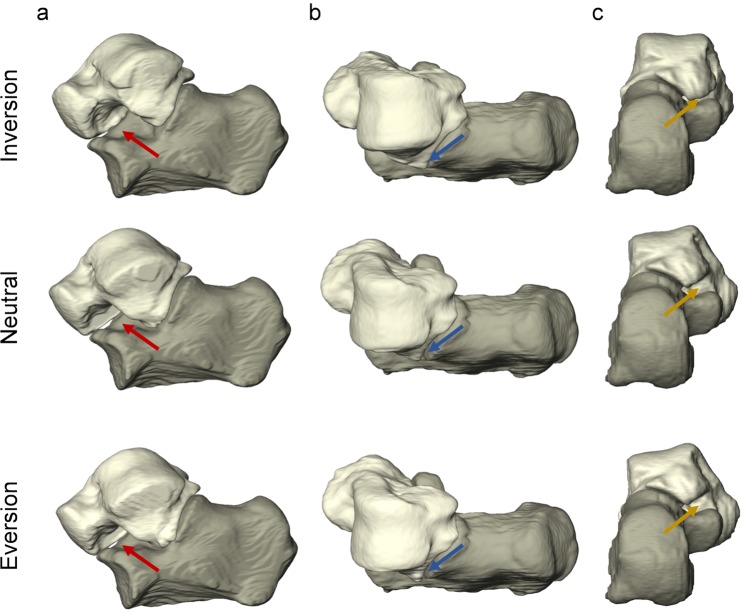
3D rendering of the subtalar joint in inverted, neutral and everted positions in the left foot of one subject from a (**a**) lateral, (**b**) superior and (**c**) posterior view. The calcaneus is shown fixed to demonstrate the relative motion of the talus in the three configurations. Lateral opening and closing of the sinus tarsi can be observed in inversion and eversion relative to neutral position, respectively (red arrows). The lateral tubercle in the talus rotates towards the sustentaculum tali from inversion to eversion (yellow arrows), whereas the lateral malleolar surface approaches the sinus tarsi (blue arrows).

**Figure 3:**
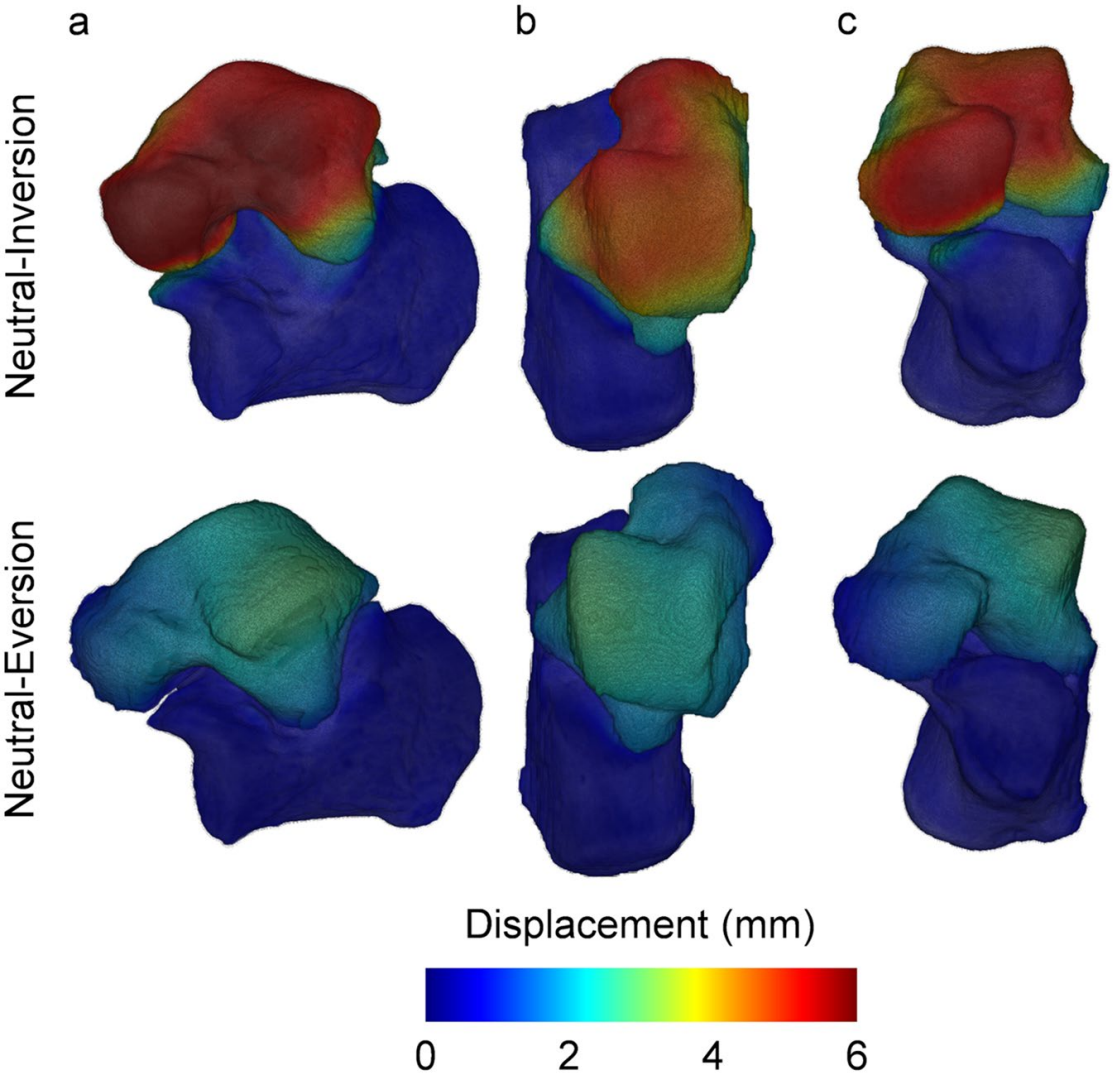
Local 3D displacements in talus and calcaneus from neutral to inversion and neutral to eversion position in the left foot of one subject from an (**a**) lateral, (**b**) superior and (**c**) anterior view as computed using DVC. Higher displacements in the talus can be observed in the inverted foot compared to the everted one.

**Figure 4:**
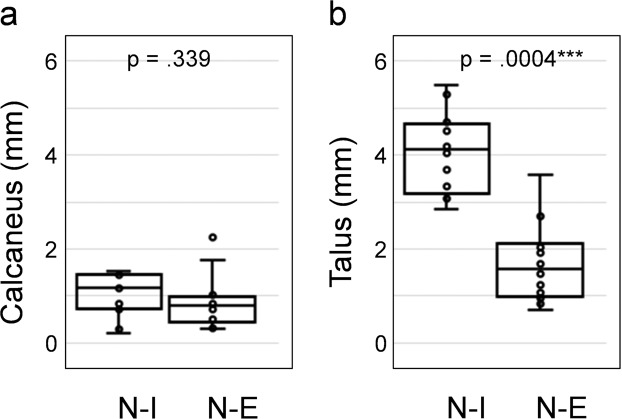
Boxplot distribution of the mean displacements over the entire bones for both (**a**) calcaneus and (**b**) talus from neutral-inversion (N-I) and neutral-eversion (N-E) positions. p-values from two-sided Wilcoxon signed-rank test are reported in each plot.

Motion of the subtalar joint was captured from weight-bearing clinical CT images acquired in three different positions: inversion, neutral and eversion. The position of the talus relative to the fixed calcaneus in such poses after segmentation of both bones and rigid registration of the calcaneus demonstrates the lateral opening of sinus tarsi in the inverted foot and closing in the everted foot as shown in Fig. 2. The DVC-computed displacement field (Fig. 3) showed lower motion of the calcaneus compared to the talus as expected from the previously performed calcaneus registration. Higher displacements could be identified in the talus from neutral to inversion positions compared to the neutral to eversion. Highest displacements in the inverted foot were localised mainly in the talar head, whereas in the everted foot higher displacements were observed in the lateral-superior talus. The mean displacement in the calcaneus was found to be around or below 1.00 mm for all subjects, with only one foot exceeding 2.00 mm (Fig. 4a). The mean talus displacements were statistically different (p = 0.0004) from neutral to inversion position compared to neutral to eversion (Fig. 4b). In both motions, mean displacements over the entire calcaneus ranged from 0.30 mm to 2.24 mm and mean displacements over the talus between 0.72 mm to 5.49 mm among all subjects. No significant differences were found between left and right feet displacements in both talus and calcaneus (Fig. S1, Supplementary material).

### Helical axis

**Figure 5:**
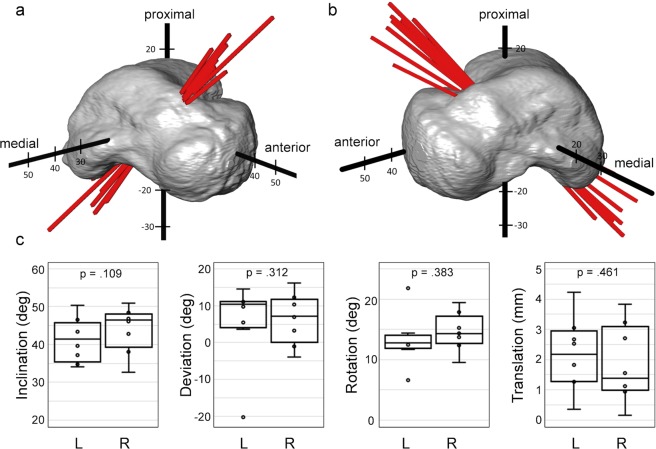
Graphic representation of the helical axis for subtalar motion from inversion to eversion in the (**a**) left and (**b**) right feet of the eight healthy subjects. To highlight the differences in axis orientation, all helical axes are grouped by overlaying a representative talus-based XYZ-coordinate system of the subjects and passing through the centre of mass of the talus (origin of the XYZ-coordinate system). (**c**) Boxplot distribution of helical axis parameters (inclination angle, deviation angle, rotation and translation) for the subtalar joint motion from inversion to eversion in both left (L) and right (R) feet. Data outliers (above/below the whiskers) do not belong to the same subject. p-values from two-sided Wilcoxon signed-rank test are reported in each plot.

The helical axes representing the range of motion of the subtalar joint between inversion and eversion positions were consistent in the group of eight subjects for both feet (Fig. 5a,b), with a predominant medially oriented direction. The inclination angle of the helical axis showed a good consistency (Fig. 5c) with a standard deviation ranging from 5.42° for the left feet cohort to 5.65° for the right one and an average angle of 41.2° and 44.1° in left and right feet, respectively. The mean deviation angle of the helical axis was found to be 5.8° for the left and 6.4° for the right feet, with higher standard deviation in the left cohort (10.4°) compared to the right one (6.3°), due to the presence of one outlier (Fig. 5c). The rotation for inversion to eversion ranged from 6.6° to 21.2° in all subjects and good consistency in the total translation was found in both left and right feet with standard deviation below 1.2 mm (Fig. 5c). No statistically significant differences between right and left side were found for any of the helical axis parameters measured.

### Centre of rotation

**Figure 6:**
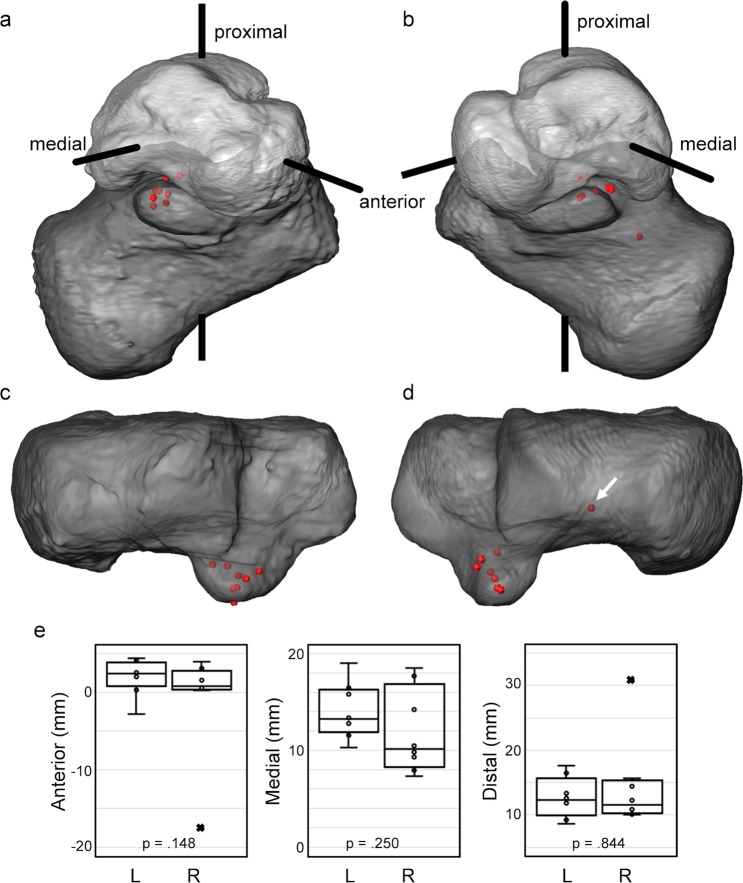
Graphic representation of the centres of rotation for subtalar motion from inversion to eversion in the (**a**,**c**) left and (**b**,**d**) right feet of the eight healthy subjects. (**a**,**b**) To highlight the differences in the centres of rotation location, these are grouped by overlaying a representative talus-based XYZ-coordinate system with the calculated centres for all subjects. (**c**,**d**) Superior view of the calcaneus with overlaying centres of rotation. (**e**) Boxplot distribution of centre of rotation location (anterior, medial and distal) for the subtalar joint motion from inversion to eversion in both left (L) and right (R) feet. Data outliers marked with × belong to the same subject, and it is identified in (**d**) (white arrow). p-values from two-sided Wilcoxon signed-rank test are reported in each plot.

The calculated centres of rotation of the relative motion of the talus around the calcaneus showed good agreement for all subjects (Fig. 6a,b). These were mainly located between the medial and posterior talus facets in the sustentaculum tali at a distance ranging from 13.8 mm to 36.2 mm from the centre of mass of the talus (origin of the XYZ-coordinate system). The standard deviation of the centre of rotation position in the left feet was lower (2.1 mm, 2.6 mm and 2.9 mm in anterior, medial and distal orientations, respectively) compared to the right feet cohort (4.1 mm, 6.5 mm, 6.8 mm in anterior, medial and distal orientations, respectively) as a result of an outlier (Fig. 6c). However, no significant differences between right and left side occurred.

**Figure 7:**
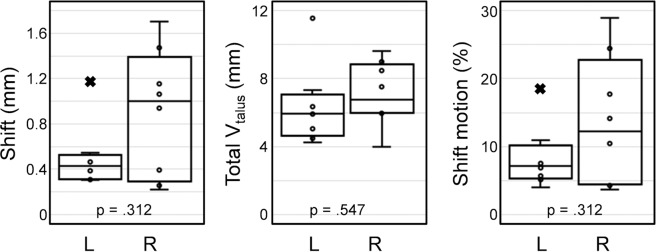
Boxplot distribution of the shift of the centre of rotation, total range of talus displacement and percentage shift motion in both left (L) and right (R) feet. Data outliers marked with × belong to the same subject. p-values from two-sided Wilcoxon signed-rank test are reported in each plot.

A comparison of the shift of the calculated centre of rotation from the neutral to the rotated positions and the total range of talus displacement computed using DVC is shown in Fig. 7. The mean shift of the centre of rotation was larger for right feet (0.90 mm) compared to the left ones (0.50 mm), but no significant difference was observed. The average range of talus displacement varied from 6.4 mm to 7.1 mm from left to right side, respectively and only exceeded 10 mm (outlier) for one subject. The percentage shift motion from inversion to eversion positions was more restricted in the left feet (8.3 ± 4.3%) than in the right cohort (13.5 ± 9.0%). In all cases, the shift of the centre of rotation did not represent more than 30% of the total range of talus displacement.

The outliers identified in the computation of helical axis (Fig. 5c), centre of rotation (Fig. 6e) and shift of centre of rotation (Fig. 7c) corresponded to different subjects.

## Discussion

This is the first study to use weight-bearing clinical CT to evaluate subtalar joint helical axis from inversion to eversion positions in healthy standing subjects. This novel approach combined 3D images to measure displacements of the bones via digital volume correlation (DVC) under various loaded states *in vivo* and proved to be robust, showing consistency of the helical axis parameters for the subtalar joint among subjects in different foot positions.

It was shown that the helical axis of the subtalar joint runs from postero-lateral-inferior to antero-medial-superior (Fig. 5a,b), with an average angle of inclination of 42.6° and deviation of 6.1° in a full weight-bearing position. This is consistent with literature, which showed a range of inclination from 29° to 51° and a range of declination from 5° to 29°. Table 1 shows the results from other studies in which there was either no load or an attempt at simulating loads, although it is very difficult to simulate loads of 300-400 N to reflect the full weight-bearing situation. The wide variance reported using other methods including anatomical landmarks, motion tracking or MRI, may reflect methodological inaccuracies, due to the lack of external landmarks on the talus. Two studies that used CT scans had less variation in the inclination and deviation angles and were closer to the findings of this paper^35,48^. This may result from the subject-specific talus-based coordinate system, defined differently from previous studies, in which the helical axis was defined based on the anatomical planes of the foot^1^. A coordinate system based on the geometric principal axis minimise inaccuracies related to the manual selection of anatomical landmarks based on 2D projections of MRI, CT or X-rays^29,32^ to define the anatomical planes of the foot and, consequently, the variability among subjects is reduced.

**Table 1:** Inclination and deviation of subtalar joint helical axis and comparison with published literature datasets. Data are reported as average (±standard deviation). (n = number of specimens).

	Inclination	Deviation	n	Method
(Manter^63^)^a^	42°(±4.5°)	16°(±4°)	16	*Ex vivo*, anatomical landmarks
(Root *et al*.^64^)^a^	41°(±8.2°)	17°(±5.2°)	22	*Ex vivo*, anatomical landmarks
(Close *et al*.^28^)	42°(—^b^)	16°(—^b^)	8	*In vivo*, anatomical landmarks
(Isman and Inman^20^)^a^	41°(±9°)	23°(±11°)	46	*Ex vivo*, anatomical landmarks
(van Langelaa^65^)^a^	41°(±8°)	22°(±7.1°)	10	*Ex vivo*, anatomical landmarks
(Lundberg and Svensson^18^)	29°(±15°)	23°(±17.4°)	8	*In vivo*, anatomical landmarks
(van den Bogert *et al*.^27^)	35°(±4.8°)	18°(±16.2°)	14	*In vivo*, anatomical landmarks
(Leardini *et al*.^66^)	53°(±4.2°)	39°(±3.9°)	6	*Ex vivo*, anatomical landmarks
(Payne *et al*.^67^)	—^b^	9°(±4.2°)	47	*In vivo*, footprint
(Arndt *et al*.^17^)	34°(±2°)	20°(±3.7°)	2	*In vivo*, anatomical landmarks
(Lewis *et al*.^68^)	38°(±6.2°)	21°(±3.6°)	6	*Ex vivo*, anatomical landmarks
(Beimers *et al*.^35^)	51°(±4.3°)	5°(±7.8°)	20	*In vivo*, simulated load CT
(Goto *et al*.^34^)	40°(±8°)	—^b^	4	*In vivo*, simulated load MRI
(Sheehan^16^)	Variable	Variable	25	*In vivo*, simulated load MRI
(Fassbind *et al*.^33^)	43°(±6°)	20°(±5°)	10	*In vivo*, simulated load MRI
(Parr *et al*.^48^)	45°(±3.8°)	5° (±3.4°)	58	*Ex vivo*, CT reconstruction
(Montefiori *et al*.^32^)	41°(±14°)	27°(±9°)	38	*In vivo*, MRI reconstruction
This study	43°(±5.7°)	6°(±8.6°)	16	*In vivo*, full weight-bearing CT

^a^Standard deviation calculated from reported range of motion (SD = Range/4).

^b^Data not reported.

The current results have shown that the average range of motion of the subtalar joint amounts to 13.9° ± 3.5° during inversion-eversion motion of the foot. Other studies that have estimated subtalar rotation using a simulated load bearing scan have shown rotations ranging from 15.1° ± 9.7°^16^ to 37.3° ± 5.9°^35^. Difference may reflect the more accurate measurement of subtalar rotation in subjects that were weight-bearing, whereby the combination of tensile loading of the plantar ligaments and compression loading of all the joints of the feet creates a more tightly joined and stable architecture when compared to non-weight-bearing conditions^19^.

The use of DVC with images from clinical CT remains partially unexplored^49,50^. Here, DVC was introduced for the first time to quantify, *in vivo*, subtalar joint displacements in weight-bearing conditions. Not only the definition of the helical axis parameters (inclination and deviation angles, rotation and translation) could be defined but, in addition, the 3D full-field displacement of the talus motion around the calcaneus (Fig. 3) was measured. To assess the accuracy of the proposed DVC methodology, the inclination and deviation angles of the helical axis of the subtalar joint were also measured (Fig. S2, Supplementary Information) using previous defined methodologies based on rigid image registration^35^ and 3D bone reconstruction^48^. The use of morphological fitting of an articular surface^32,48^ to define the helical axis produced higher variability of the measured inclination and deviation angles (43.0° ± 12.9°and 5.5° ± 13.8°, respectively). This can be explained by the need to manually define calcaneal and sustentaculum facets, which unavoidably introduce operator-dependent variability. Similar values were found by rigidly registering the obtained CT images (42.0° ± 4.6° and 6.0° ± 9.5°, for inclination and deviation angles). The advantage of using DVC before a rigid registration procedure relies on its ability to track the displacement of the internal bone structure. Therefore, the local displacements can be quantified instead of the global rotation and translation. Furthermore, not just the boundary of the bones^35^ (whose definition may be subjected to segmentation errors) is considered in the matching procedure between two different foot positions, but the inner bone structure can be analysed. This shows the large potential of DVC to better understand foot and ankle motion *in vivo* when combined with weight-bearing clinical CT. Although not the objective of the present work, full-field strain distribution in bone could be quantified, allowing a novel diagnostic tool for foot and ankle deformities.

In conditions such as post-traumatic arthritis, or symptomatic talocalcaneal coalition, posterior tibial tendon dysfunction, isolated subtalar joint instability or inflammatory arthritis, surgical fusion of the subtalar joint is currently the gold standard treatment^51–53^. However, fusion of hindfoot joints has been shown to transfer the stresses to adjacent joints leading to progressive arthritis and symptoms in these joints^10–13^. Any previous attempts to replace rather than fuse the subtalar joint have been based on two conforming surfaces that allowed sliding and translation and failed to replicate the somewhat constrained rotation of the talus on the calcaneus that is seen *in vivo*^14^. In all cases the patients had pain and probable clinical instability, leading to abandonment of the procedure^14^.

The second goal of our study was to establish whether the centre of rotation of the subtalar joint, as a central pivot around which the talus rotates, could be determined. The centre of rotation of other joints, such as the hip, knee and ankle, have been previously calculated using anatomical landmarks^21,22,24,25^ or dual fluoroscopic imaging^23^; however, the calculation of the centre of rotation for the subtalar joint has never been addressed, which can be partially explained by the difficulties in tracking subtalar joint motion *in vivo*. A subject-specific method of locating the centre of rotation based on a sphere fitting approach of the centre of mass of the talus has been herein proposed and it was shown that the centre or cluster of centres appears to be in the region of the middle facet of the subtalar joint (Fig. 6). The ability of precisely determining the centre of rotation might help to inform the development of a joint replacement that can replicate *in vivo* kinematics of the subtalar joint which hitherto has never been successful.

The reconstruction of the rotational motion of the talus around the calcaneus (Supplementary Videos 1 and 2) may indicate that subtalar joint motion is mainly a rotation around the helical axis centred around the middle facet of the subtalar joint. Whilst some translation along the helical axis was identified, as demonstrated by a shift in the centre of rotation of 0.7 mm ± 0.46 mm which represents 10.9% (±7.5%) of the total range of talus displacement, it is hypothesised that this reflects the elasticity of the soft tissue restraints, namely the strong subtalar ligaments. Following severe ankle injury, it is not uncommon to also injure the subtalar joint ligaments leading to both ankle and subtalar joint instability^2,3,54^. Indeed, subtalar joint instability appears to be more frequent than is generally assumed^2^. Augusto *et al*. (2019) used 4D CT to assess differences in talus/calcaneus relative angles and distances during motion in the non-weight-bearing setting between healthy individuals and patients with subtalar joint stiffness and instability. They mainly looked at relative kinematic measurements of the talus and calcaneus in the virtual reconstructed radiographs (2D) from conventional clinical CT images^37^. However, 3D motion (i.e. rotation and translation) of the subtalar joint was not addressed. The potential of 3D volumetric data was also not exploited, as it has been performed in the present study.

Various limitations of this study should be acknowledged. First, a limited number of subjects (n = 8) participated in the experiment; therefore, generalisation and extrapolation of the location of the centre of rotation of the subtalar joint in healthy individuals warrants further analysis. Nevertheless, high consistency has been shown among subjects. Another limitation is related to the use of standardised wedges to image inversion and eversion positions for all subjects. Even though each subject was asked to place their feet on the wedges so that they felt it was the maximal inversion or eversion they could achieve, this may not represent the total range of motion of the subtalar joint. Last, this study quantified subtalar joint rotation and translation from static weight-bearing CT images acquired at different degrees of rotation. This acted as a surrogate for motion, but the dynamic motion between the talus and calcaneus was not addressed. While 4D CT or the combination of dual fluoroscopic imaging with CT can provide dynamic information of subtalar joint movement, motion artefacts occurring during imaging acquisition could affect 3D image reconstruction and consequently, DVC computation. In addition, it may be difficult to obtain ethical approval given the much greater radiation doses that this would involve. Nonetheless, coupling weight-bearing static and dynamic CT for the evaluation of subtalar joint kinematic could lead to a better identification and description of subtalar joint pathologies.

Further research will be needed to establish how the normal subtalar motion changes under load, when the soft tissue restraints are pathological such as following ligamentous injury or in patients with lax ligaments, or indeed in the presence of arthritis and stiffness. The methodology described in this paper could also be used to assess hindfoot motion after surgery paving the way for an improved understanding of the outcomes of surgery which hitherto have been next to impossible to evaluate in the clinical setting.

In conclusion, this study demonstrated the feasibility of a 4D image-based patient-specific procedure to evaluate subtalar joint motion with full weight-bearing clinical CT in healthy subjects. The quantification of the 3D full-field displacement of the subtalar joint during inversion-eversion motion using DVC provided a novel tool not only to measure the helical axis *in vivo* but also to give a better understanding of the relative motion at the subtalar joint under physiological loading. It was determined that the helical axis of the subtalar joint runs from postero-lateral-inferior to antero-medial-superior with an average angle of inclination of 42.6° and a deviation of 6.1° in a full weight-bearing position. It has also been shown that the centre of rotation is located in the region of the middle facet of the subtalar joint with on average up to 11% translation in normal subjects reflecting the viscoelasticity of the normal subtalar ligaments. It is hypothesised that the translational component is likely to be greater in patients with ligamentous disruption and hypermobility. Any joint replacement developed to replace this joint will need to replicate normal motion and incorporate appropriate constraints to prevent clinical instability. 4D image-based analysis shows promise to assess changes in motion and kinematics in normal and pathological cases, helping to direct better clinical understanding and future treatments.

## Methods

### Subjects and image acquisition

**Figure 8:**
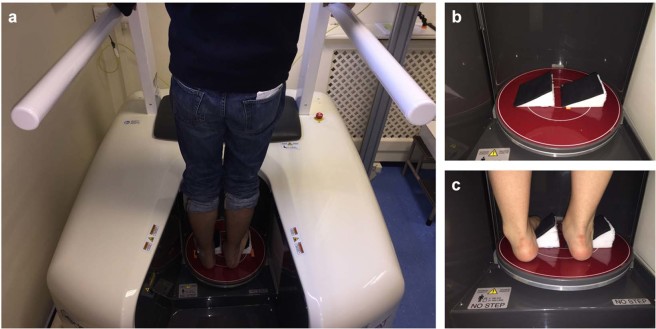
Bilateral weight-bearing PedCAT-CT. An X-ray source and a flat-panel detector on opposite sides rotates horizontally around the foot. (**a**) Subject positioned in bipedal standing position in pedCAT during scan. (**b**) Wedges placed in pedCAT platform to allow for eversion and inversion position of the feet. (**c**) Subject standing on the wedges with right foot in eversion and left foot in inversion configuration.

The study received ethical approval from the London Ethics Committee (16/LO/025) and all methods were performed in accordance with the relevant guidelines and regulations. Eight healthy asymptomatic volunteers (5 males and 3 females with an age ranging from 30 to 57 years old) participated in this study following receipt of informed consent. Each subject walked into a PedCAT (Curvebeam, Warrington, USA) standing CT scanner and positioned into the centre of two rings on a baseplate (Fig. 8a). Although there are handles to balance, the subject was asked to apply full and equal weight onto both feet in a neutral position for the initial scan. The subject then stepped out whilst two custom wedges (angle of inclination of 30°, maximum height of 16 cm) were placed into the scanner covered in a rough surface to prevent slipping (Fig. 8b). The subject was then asked to stand onto the wedges (Fig. 8c) with one foot inverted (high arch position) and the other everted (flat foot position). Each subject moved their foot position on the wedges until they obtained what they perceived as maximum inversion or eversion for themselves as judged by starting to feel uncomfortable. After a scan was performed, the subject was asked to turn around by 180° so that the inversion and eversion were reversed, and a further scan was performed ensuring for each subject a neutral, everted and inverted subtalar joint position. For each subject, both feet and ankle were scanned over the entirety 30 cm superior from the standing platform. The final voxel size achieved was 370 μm and the total scanning time per subject was 144 seconds (48 seconds per scan). After image acquisition, X-ray projections were 3D reconstructed allowing multi-planar view of the subtalar joint in the three configurations (Fig. 9.1). The total radiation dose to each subject following all three scans was estimated to be 1.9 µSv, which is less than a single conventional CT^55^.

### Image post-processing

**Figure 9:**
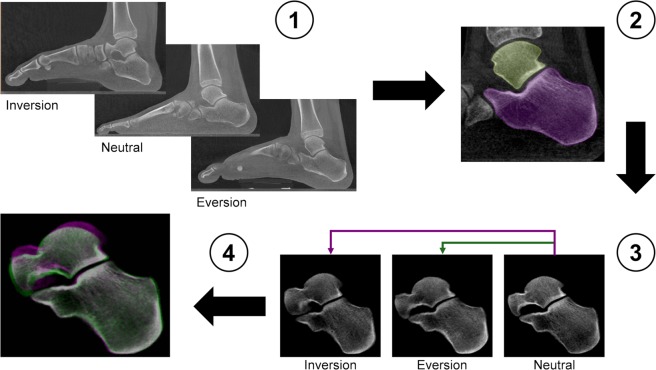
Workflow of the image post-processing. (1) Weight-bearing clinical CT images of the entire foot in inversion, neutral and eversion positions. (2) Semi-automatic active contour segmentation of the individual bone in the subtalar joint. (3) The calcaneus in the rotated positions (inversion/eversion) was rigidly register with the corresponding calcaneus in the neutral position. (4) Subtalar joint after rigid registration of the calcaneus. Pink represents inversion and green eversion positions. When pixels of the three configurations match, they displayed the colour grey. After registration, the calcaneus of the three images is perfectly aligned and the relative talus motion can be assessed.

Image post-processing prior to quantitative analysis of subtalar joint kinematics was performed according to a fixed workflow (Fig. 9). Bone segmentation of the talus and calcaneus was performed by a semi-automatic active contour algorithm (Avizo 9.4, Thermo Fisher Scientific, Massachusetts, USA) in the neutral, eversion and inversion configurations. From a number of specified seed points in each bone (i.e. talus, calcaneus) a boundary front evolves. The active contour algorithm computes the position of the front at all times until the outline of the bone structure is identified (Fig. 9.2).

For quantitative analysis of the subtalar joint kinematics, the motion of the talus relative to the calcaneus between neutral-inversion and neutral-eversion positions were computed. Firstly, a rigid registration was performed (ImageJ^56^) to align the calcaneus in inversion and eversion positions with the neutral reference configuration (Fig. 9.3). Registration was performed minimising the Euclidian distance (corresponding to the square root of summed squares of voxel intensity differences) between the reference (i.e. neutral) and the target (i.e. inversion/eversion) image, using an iterative optimisation algorithm (conjugate direction search^57^). Both translation and rotation parameters for each bone were computed in order to find an optimal fit in the three dimensions (Fig. 9.4). Then, the obtained transformation was applied to the talus in both inversion and eversion positions. Finally, for each individual talus and position, a local right-hand rule XYZ-coordinate system was defined based in the geometric principal axes of the talus of the subject as in^35^. The origin of the talus-based coordinate system was placed in the centre of mass of the talus, which was calculated based on the grey-scale pedCAT images (Matlab 2017b, MathWorks Inc., Natick, USA). The positive major principal axis (X-axis) was directed anteriorly, the positive second principal axis (Y-axis) medially and the positive third principal axis (Z-axis) proximally.

### Calculation of subtalar joint axis

Digital volume correlation (DaVis 8.4, LaVision, Goettingen, Germany) was used to compute the displacement field of both talus and calcaneus from neutral to inversion and neutral to eversion positions. DVC is a cross-correlation method operating on the intensity values (grey-scale) of the 3D reconstructed CT images. In essence, the measurement volume is divided into smaller sub-volumes and the contrast (grey-scale) pattern is then tracked from reference (neutral position) to deformed (inversion/eversion position) state as a discrete function of the grey-levels. The operating principles of DVC methods have been extensively reported elsewhere^58,59^. DVC computation was conducted using a multi-pass scheme with a final sub-volume of 8 voxels (2.96 mm^3^), reached via successive (predictor) passes using sub-volumes of 40, 32, 24 and 16 voxels. To quantify the level of uncertainties of the DVC measurements, which is associated to imaging conditions, image post-processing and sub-volume size of the rigidly registered calcaneus images were used. In an ideal repeated CT image, the displacements could be considered null; however, in this real experiment the actual displacements were affected by the actual motion of the joint in the different positions and the registration procedure. Therefore, the accuracy of the computed displacement (mean values) could not be evaluated and only the precision (standard deviation) was assessed^60^. Precision of the DVC-computed displacements ranged from 0.25 to 0.02 mm (0.66 to 0.07 voxels) among all pair of images analysed. Only sub-volumes fully included within the talus or calcaneus were considered for the displacement field calculation to avoid inaccuracies in the segmentation procedure that may affect the surface of both talus and calcaneus. In addition, a threshold for the correlation coefficient was set to 0.5 and just sub-volumes exceeding such threshold were analysed.

The relative displacements of the talus, with respect to the mean calcaneus displacement, from the neutral position at an instant time *t* = *0* to inversion/eversion position at time *t* were then computed [Eq. (1)]

1 $$\,{}_{0}{}^{t}\Delta {\boldsymbol{V}}={{\boldsymbol{V}}}_{talus}-{\overline{{\boldsymbol{V}}}}_{calcaneus}$$

As the talus moves from neutral to inversion/eversion, the position of each defined sub-volume within the talus can be defined as [Eq. (2)]

2 $$\,{}_{0}{}^{t}\Delta {\boldsymbol{X}}={{\boldsymbol{X}}}_{talus,0}+{}_{0}{}^{t}\Delta {\boldsymbol{V}}$$

The position of each sub-volume at both rotated positions is then used as a landmark to determine the motion of the talus by tracking such landmark positions using least-squares methods^61^. A singular value decomposition of the matrix derived from the position of the sub-volumes is used to determine the transformation matrix (containing both rotation and translation), which expresses the movement of the talus from inversion to eversion positions. Such movement can also be considered the result of a rotation through an angle about the helical axis and a translation along that axis. Thus, the helical axis parameters for motion of the talus relative to the calcaneus, between inversion and eversion positions, were computed.

To define the orientation of the helical axis in the XYZ-coordinate system, its inclination and deviation angles were calculated for each testing subject and foot. The inclination angle is defined as the angle between the helical axis and the XY-plane and the deviation angle is defined as the angle between the projection of the helical axis on the XZ-plane and the X-axis as in^35^. The transformation matrix and helical axis parameters were computed with mathematical routines developed in Matlab software (Matlab R2017a, The MathWorks Inc., Natick, USA).

### Calculation of centre of rotation

**Figure 10:**
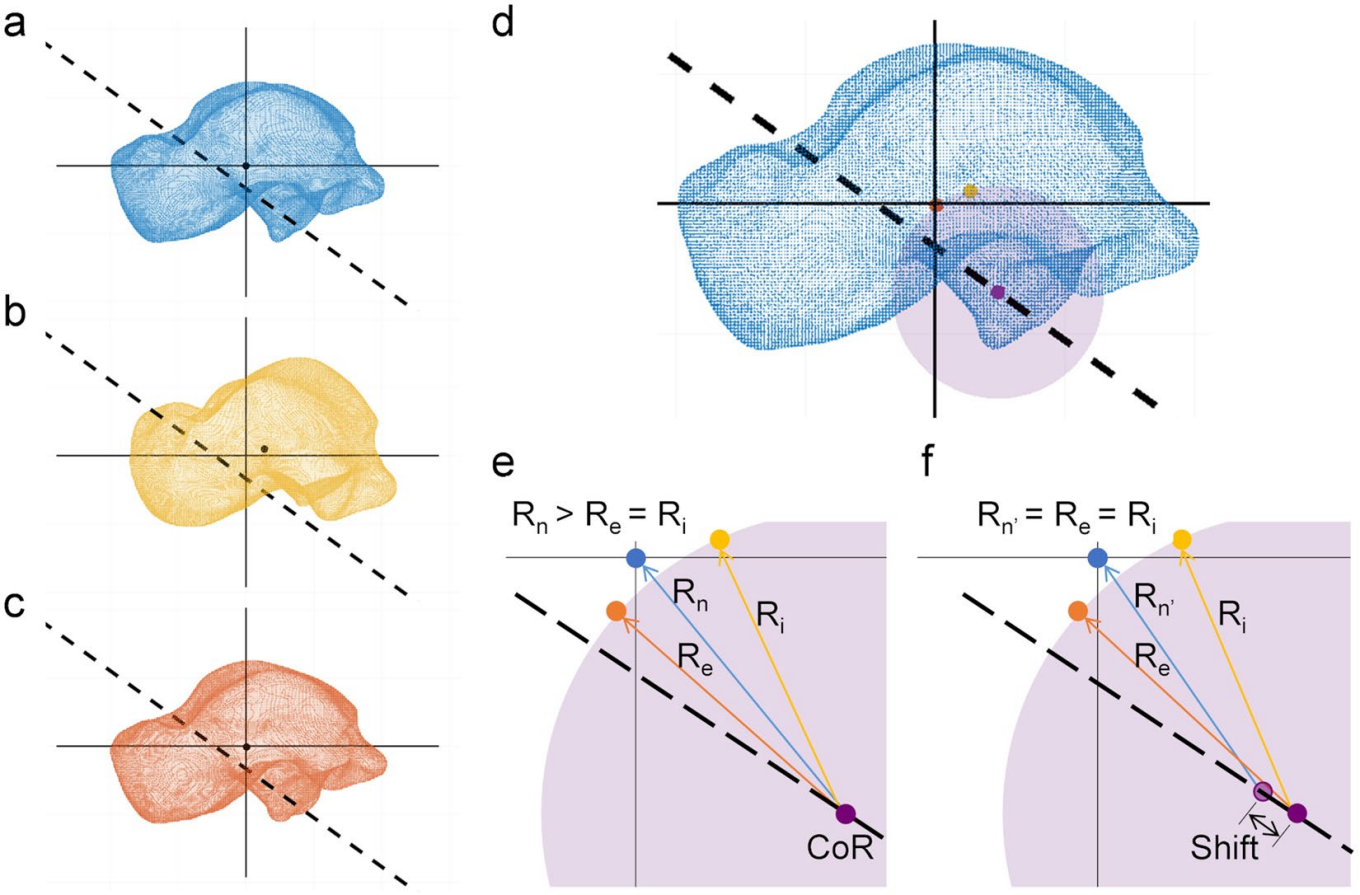
Schematic representation for the calculation of the centre of rotation and its translation on the helical axis. The centre of mass of the talus in (**a)** neutral, (**b**) inversion and (**c**) eversion positions was computed based on the grey-intensity value of the PedCAT images. (**d**) A sphere-fitting approach was used to define the parameters of a sphere (purple) with centre on the helical axis (dashed line) and with the centre of mass of the talus in the rotated configurations (orange and yellow dots) on its surface. The centre of rotation of the talus relative to the calcaneus was determined as the centre of such sphere (purple dot). (**e**) The distances from the centre of rotation (CoR) to the centre of mass of the talus in neutral (R_n_), inversion (R_i_) and eversion (R_e_ = R_i_) positions were computed. (f) The translation of the centre of rotation (i.e. shift) was defined as the difference between such distances (Shift = R_n_ − R_n′_).

The centre of rotation of the talus relative to the calcaneus was determined as the central pivot of the talus at the subtalar joint axis. A sphere fitting approach^21^ was used, assuming the centre of rotation to be stationary and lying on the helical axis^62^ (Fig. 10). It was hypothesised that the centre of mass of the talus moves on the surface of a sphere with specific radii around a common calcaneus centre. Thus, the trajectory of the centre of mass of the talus during subtalar joint motion, from inversion to eversion position, describes the surface of such sphere with the centre of rotation as the centre of the sphere and the helical axis passing through such centre. The centre of mass of the talus in neutral, inversion and eversion configuration were calculated based on the grey-intensity value of the pedCAT images (Fig. 10a–c). The parameters of the sphere (centre and radius) with centre in the helical axis and the calculated centres of mass in the rotated positions on its surface were computed in Matlab software for each subject/foot (Fig. 10d).

To evaluate the translation of the centre of rotation on the helical axis, the distance from the centre of mass of the talus in neutral position to the centre of rotation (R_n_) was computed and compared to the radius of the fitted sphere (i.e. distance from the centre of rotation to the centre of mass in inversion/eversion position, R_e_/R_i_) (Fig. 10e). The difference between such distances was defined as the shift of the centre of rotation (Fig. 10f). Any translation of the centre of rotation, related to the viscoelasticity of the subtalar ligaments, was then compared to the total range of DVC-computed talus displacement from inversion to eversion configuration [Eq. (3)]

3 $$\begin{array}{c}Total\,{V}_{talus}=\mathop{\max }\limits_{t}{V}_{talus}-\mathop{\min }\limits_{t}{V}_{talus}\end{array}$$

Finally, the ratio of translation of the centre of rotation relative to the total range of talus displacement was evaluated and defined as a shift motion.

### Statistics

Quantitative variables are reported as mean ± standard deviation and represented as median, range, 95% confidence intervals (CIs) and outliers on boxplots (Microsoft Excel 2016, Redmond, USA). The asymmetry between the left and right foot helical axis parameters and centre of rotation as well as differences between neutral-inversion and neutral-eversion displacement were compared using a two-side Wilcoxon signed rank test (*α* = 0.05) in Matlab software.

## Supplementary information

Supplementary Information.

Video S1.

Video S2.

## Data Availability

The datasets generated and/or analysed during the current study are available from the corresponding author on reasonable request.
