# Supplementary material for: Centre of Rotation of the Human Subtalar Joint Using Weight-Bearing Clinical Computed Tomography
Source: Sci Rep. 2020 Jan 23;10:1035. doi: 10.1038/s41598-020-57912-z (PMC6978465; doi:10.1038/s41598-020-57912-z)

Supplementary Information for

Centre of Rotation of the Human Subtalar Joint Using Weight-Bearing Clinical Computed Tomography

Marta Peña Fernández[1*], Dorela Hoxha[1], Oliver Chan[2], Simon Mordecai[2], Gordon Blunn[3], Gianluca Tozzi[1], Andy Goldberg[2,4,5]

Affiliations:

1. Zeiss Global Centre, School of Mechanical and Design Engineering, University of Portsmouth, Portsmouth, PO1 3DJ, UK.
2. Institute of Orthopaedics & Musculoskeletal Science, Royal National Orthopaedic Hospital, Stanmore, HA7 4LP, UK
3. School of Pharmacy and Biomedical Sciences, University of Portsmouth, Portsmouth, PO1 2DT,UK.
4. MSK Lab, Faculty of Medicine, Department of Surgery & Cancer, Imperial College London, Level 2, Faculty Building, South Kensington Campus, London SW7 2AZ, UK.
5. The London Ankle & Arthritis Centre, The Wellington Hospital, Wellington Place, London NW8 9LE, UK.

Supplementary Legends

Figure S1. DVC-computed displacements. Boxplot distribution of the mean displacements over the entire bones for both (a) calcaneus and (b) talus from neutral-inversion (N-I) and neutral-eversion (N-E) positions in both feet. Data outliers are marked with ○. p-values from two-sided Wilcoxon signed-rank test are reported in each plot.

Figure S2. Comparison of helical axis parameters. Boxplot distribution of helical axis parameters, (a) inclination angle and (b) deviation angle, for the subtalar joint motion form inversion to eversion position in the entire cohort computed using different methodologies. The introduced methodology, digital volume correlation (DVC), is compared to previous reported methods such as morphological fitting of the articular surfaces (Montefiori et al., 2019; Parr, Chatterjee, & Soligo, 2012) and rigid registration of the CT data (Beimers et al., 2008). Morphological fitting produced higher variability of the measured angle whereas rigid registration showed similar results to DVC method. p-values from two-sided Wilcoxon signed-rank test are reported in each plot.

Supplementary Video 1. Simulation of talus rotation around the computed centre of rotation for the subject showing lowest shift along the helical axis. The calcaneus is shown fixed while the talus rotates. Both extreme positions, inversion (blue) and eversion (red) are overlay to the neutral (grey) rotated talus.

Supplementary Video 2. Simulation of talus rotation around the computed centre of rotation for the subject showing highest shift along the helical axis. The calcaneus is shown fixed while the talus rotates. Both extreme positions, inversion (blue) and eversion (red) are overlay to the neutral (grey) rotated talus.

Supplementary Figure S1

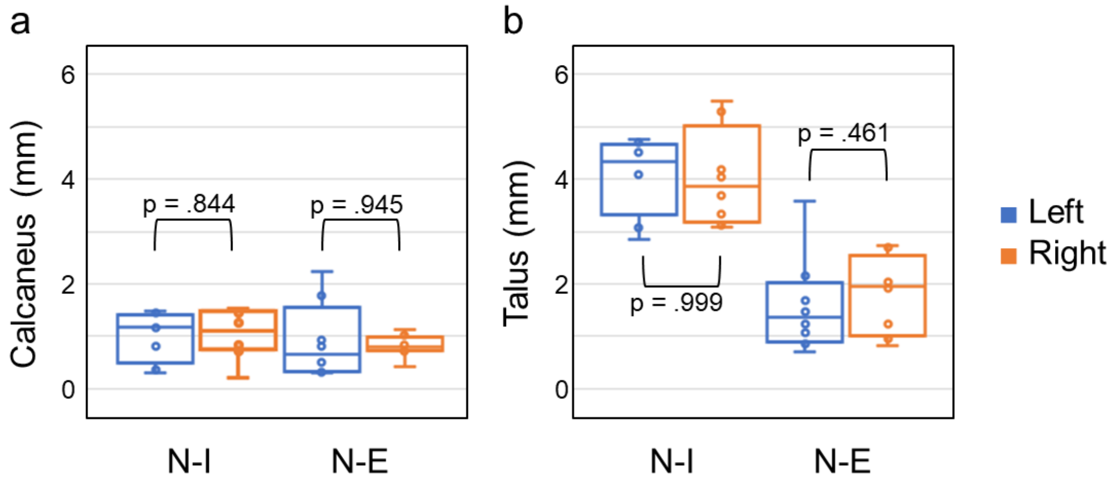

Supplementary Figure S2

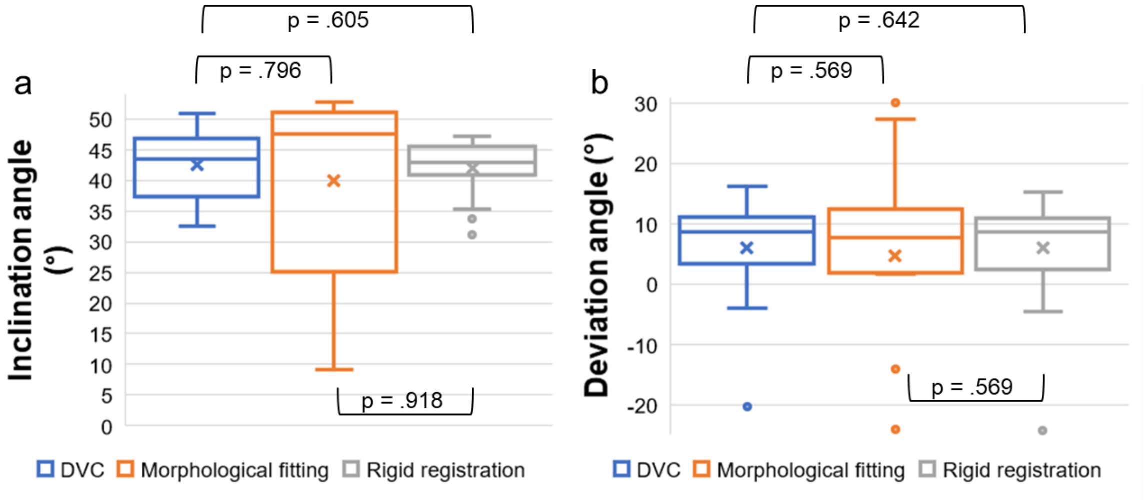

Supplement: Supplementary file 1 — Supplementary Information. [file 41598_2020_57912_MOESM1_ESM.pdf]
